# Oral maintenance therapy using apatinib combined with S-1/capecitabine for esophageal squamous cell carcinoma with residual disease after definitive chemoradiotherapy

**DOI:** 10.18632/aging.202652

**Published:** 2021-03-10

**Authors:** Dongmei Chi, Baoqing Chen, Suping Guo, Kunhao Bai, Huali Ma, Yonghong Hu, Qiaoqiao Li, Yujia Zhu

**Affiliations:** 1 Department of Radiation Oncology, Sun Yat-sen University Cancer Center, State Key Laboratory of Oncology in South China, Collaborative Innovation Center of Cancer Medicine, Guangzhou, Guangdong, P.R. China; 2 Guangdong Esophageal Cancer Research Institute, Guangzhou, Guangdong, P. R. China; 3 Department of Anesthesiology, Sun Yat-sen University Cancer Center, State Key Laboratory of Oncology in South China, Collaborative Innovation Center for Cancer Medicine, Guangzhou, Guangdong, P.R. China; 4 Department of Endoscopy, Sun Yat-sen University Cancer Center, State Key Laboratory of Oncology in South China, Collaborative Innovation Center of Cancer Medicine, Guangzhou, Guangdong, P.R. China; 5 Department of Radiology, Sun Yat-sen University Cancer Center, State Key Laboratory of Oncology in South China, Collaborative Innovation Center of Cancer Medicine, Guangzhou, Guangdong, P.R. China

**Keywords:** apatinib, S-1, esophageal squamous cell carcinoma, chemoradiotherapy, maintenance therapy

## Abstract

Background: A substantial number of patients with esophageal squamous cell carcinoma (ESCC) do not achieve complete remission after definitive concurrent chemoradiotherapy (dCRT). We performed this retrospective study to evaluate the efficacy and safety of apatinib combined with S-1/capecitabine as the oral maintenance therapy for these patients.

Methods: Thirty-nine ESCC patients with residual disease after dCRT were included. Patients were treated with apatinib combined with S-1 /capecitabine after dCRT. Efficacy, toxicity, and survival were analyzed.

Results: Of the 39 patients, 5 (12.8%) achieved a partial response and 29 (74.4%) achieved stable disease, yielding a disease control rate of 87.2%. The median progression-free survival (PFS) and overall survival (OS) were 27.5 (95%CI: 14.9 - 40.1) and 38.1 (95%CI: 31.3 - 44.8) months. Most frequent adverse events were of grade 1 to 2. Multivariate analysis revealed the occurrence of any adverse events (HR = 0.274, 95%[CI] = 0.119 - 0.630) correlated to better PFS and occurrence of proteinuria (HR = 0.108, 95%[CI] = 0.025 - 0.456) predicted better OS.

Conclusion: The oral combination therapy consisting of apatinib and S-1/capecitabine showed a tolerable toxicity profile and achieved satisfactory disease control in ESCC patients with residual disease after dCRT.

## INTRODUCTION

Esophageal carcinoma (EC) is one of the leading causes of cancer-related mortality worldwide [1]. Unlike esophageal adenocarcinoma, which is prevalent in western countries, the prevalence of esophageal squamous cell carcinoma (ESCC) is higher in China [2]. Definitive concurrent chemoradiotherapy (dCRT) is the standard alternative curative management for patients with locally advanced disease who are not eligible for surgery [3]. Despite an effective treatment response obtained from dCRT, approximately 33% of these patients may have the residual disease and develop recurrent disease within a year after complete remission [4, 5]. There is a lack of consensus on the standard therapeutic strategy for those with post-dCRT residual lesions. Indeed, local treatment with systemic chemotherapy remains the second-line therapy once progression is confirmed in most residual or recurrent cases [6]. However, increased incidence of toxicities associated with intravenous chemotherapy and inconvenience to patients has led to unsatisfactory treatment compliance and interruption of therapy. Compared to the intravenous route, an oral chemotherapeutic regimen may present lower toxicity and therefore, could be a feasible approach for maintenance therapy in patients with post-dCRT residual lesions.

An oral combination of tegafur, gimeracil, and oteracil potassium, named S-1, has been widely used in treating multiple types of cancers, especially in Japan. Upon internalization into cells, S-1 is converted into fluorouracil and shares similar anticancer properties as intravenous 5-Fu [7, 8]. Capecitabine is a fluoropyrimidine that enzymatically converts to 5-Fu by thymidine phosphorylase in tumor tissues, when the drug is administered orally, and has been approved to replace 5-Fu for the treatment of advanced esophagogastric cancer [9]. Since 5-Fu is one of the standard chemotherapeutics for ESCC, S-1 or capecitabine may potentially substitute 5-Fu in ESCC patients who cannot tolerate or refuse intravenous chemotherapy. In fact, patients with unresectable and recurrent ESCC achieved a promising response and showed minimum safety concerns with S-1/capecitabine monotherapy after failing first-line standard treatment [10]. These findings were confirmed in prospective studies that demonstrated the feasibility of S-1/ capecitabine combined with definitive radiation for treating ESCC [11].

Angiogenesis is one of the pivotal cancer hallmarks that promotes cancer growth and metastasis, including esophageal carcinoma, and is a target of biologicals such as bevacizumab, which blocks the vascular endothelial growth factor, VEGF, and drugs such as the multi-receptor tyrosine kinase inhibitors (TKIs) that target the VEGF receptor, VEGFR. Apatinib, one of several oral TKIs that has demonstrated high selectivity for VEGFR-2, and was given approval by the Chinese FDA in 2014 as one of the later-line treatments for gastric cancer [12]. Though the efficacy and safety of apatinib were demonstrated in a retrospective study of advanced ESCC patients who failed prior treatment [13], mono anti-angiogenic approaches have not been very successful in tumor control. Nevertheless, studies have now shown that a combination of anti-angiogenic drugs and chemotherapy may be beneficial [14]. For instance, Zhao et al. reported that a combination of apatinib and S-1 was effective and safe as a second-line treatment for advanced ESCC patients [15]. Based on these findings, we performed this retrospective analysis to evaluate the safety and efficacy of a combination therapy consisting of apatinib, and S-1/capecitabine as the oral maintenance therapy for ESCC patients with post-dCRT residual lesions.

## RESULTS

### Baseline clinical characteristics

Between December 2016 and December 2019, a total of 39 eligible ESCC patients were enrolled in this study. Patient demographics and tumor characteristics are listed in Table 1. The median age of patients was 61 years (range, 44 - 75), and 34 (87.2%) were male and five (12.8%) female. Patients were diagnosed with ESCC stage II (6; 15.4%) and stage III (33; 84.6%) and completed at least one concurrent chemotherapy cycle with single-agent (18%), taxol and platinum (TP) (20.5%), or platinum and fluorouracil (PF) (61.5%). The median radiation (RT) dose was 60 Gy (range, 50.4 – 64) and 20 (51.3%) patients received RT at ≥ 60Gy. After the completion of dCRT, 14 (35.9%) patients had stable disease (SD) and the remaining 25 (64.1%) achieved partial remission (PR). Seven patients were confirmed with fistula after dCRT. Primary esophageal residual disease was reported in 12 (30.7%) patients, 13 (33.3%) only had regional lymph nodes residual disease, and 14 (35.9%) had the residual disease at both sites. Ten of 39 patients were confirmed with the residual disease by biopsy pathology.

**Table 1:** Clinical characteristics.

**Characteristics**	**Patients No. (%)**
**Age (years)**	
Median(Range)	61(44-75)
>60	20(51.3)
≤ 60	19(48.7)
**Gender**	
Male	34(87.2)
Female	5(12.8)
**Tumor location**	
Cervical	6(15.4)
Upper	9(23.1)
Middle	13(33.3)
Lower	9(23.1)
Multiple	2(5.1)
**Length (cm)**	
<5	16(41.0)
≥ 5	23(59.0)
**TNM stage**	
II	6(15.4)
III	33(84.6)
**Concurrent Chemotherapy**	
Single-agent	7(18)
TP	8(20.5)
PF	24(61.5)
**Radiation Dose (Gy)**	
Median(Range)	60(50.4-64)
<60	19(48.7)
≥60	20(51.3)
**Response after dCRT**	
PR	25(64.1)
SD	14(35.9)
**Combined with fistula**	
Yes	7(17.9)
No	32(82.1)
**Residual disease sites**	
Primary lesion only	12(30.7)
Regional lymph nodes only	13(33.3)
Primary lesion combined with Regional lymph nodes	14(35.9)
**Pathological residual disease**	
Yes	10(25.6)
No	29(74.4)

### Response to treatment

The treatment regimens and patient response are listed in Table 2. Nineteen (48.7%) patients received sequential therapy regiments, which include S1/capecitabine given alone at first 1-2 cycles followed by dual therapy consisting of S1/capecitabine and apatinib for the remaining treatment cycles until disease progression or intolerable toxicities. S1/capecitabine combined with apatinib on treatment initiation was administered to 20 (51.3%) patients. S-1 was replaced with capecitabine once during the treatment course in seven (17.9%) patients. The median number of the treatment cycle is 12 (2- 31). Among those 39 patients, five patients refused the continuation of the regimen due to the intolerance of toxicities before the confirmed progression of the tumor. At the last follow-up, 15 patients are still in continuation of this maintenance treatment. None of the patients achieved complete remission (CR), while five (12.8%) achieved PR. The overall response rate (ORR) was 12.8% (5/39). Twenty-nine (29/39, 74.4%) patients achieved SD, and five (5/39, 12.8%) had progressive disease (PD), yielding a disease control rate (DCR) of 87.2%.

**Table 2:** Treatment and response.

**Maintenance therapy**	**N(%)**
S1/capecitabine alone followed by S1/capecitabine combined with Apatinib	19(48.7)
S1/capecitabine concurrent with Apatinib	20(51.3)
**No. of cycles**	
Median(Range)	12(2-31)
**Treatment response**	
PR	5(12.8)
SD	29(74.4)
PD	5(12.8)

### Toxicity

Table 3 shows the toxicity associated with treatment in the cohort. Most were grade 1 to 2 in severity and overlapped with apatinib’s toxicity spectrum. Of the 13 patients who developed secondary hypertension, 10 (25.6%) presented with grade 1 and 2, and three (7.7%) with grade 3. The second common adverse event was proteinuria, with one (2.6%) patient showing grade 4 toxicity, which disappeared rapidly after discontinuing apatinib. One (2.6%) patient presented with the hand-foot syndrome of grade 3 severity. Thus, a total of five (12.8%) patients presented with grade 3 to 4 non-hematological toxicity, and none with hematological toxicity. Other adverse events including fatigue, liver enzyme elevation, bleeding, hoarseness, and diarrhea, as well as anemia (8; 20.5%), leukocytopenia (3; 7.7%), and thrombocytopenia (3; 7.7%) were of grade 1 and 2 severity. No treatment-related hemorrhage or hemoptysis was found.

**Table 3:** Treatment-related toxicity.

	**Grade 1**	**Grade 2**	**Grade 3**	**Grade 4**
**Non-Hematological**				
Secondary hypertension	4(10.2)	6(15.4)	3(7.7)	0(0)
Proteinuria	10(25.6)	1(2.6)	0(0)	1(2.6)
Hand–foot syndrome	7(17.9)	3(7.7)	1(2.6)	0
Fatigue	4(10.3)	2(5.1)	0	0
Liver enzyme elevation	6(15.4)	0	0	0
Bleeding	2(5.1)	0	0	0
Hoarseness	1(2.6)	0	0	0
Diarrhea	1(2.6)	0	0	0
**Hematological**				
Anemia	8(20.5)	0	0	0
Leukocytopenia	2(5.1)	1(2.6)	0	0
Thrombocytopenia	3(7.7)	0	0	0

Acute toxicity was evaluated according to the Common Terminology Criteria for Adverse Events version 3.0 (CTCAE 3.0).

### Survival

**Figure 1:**
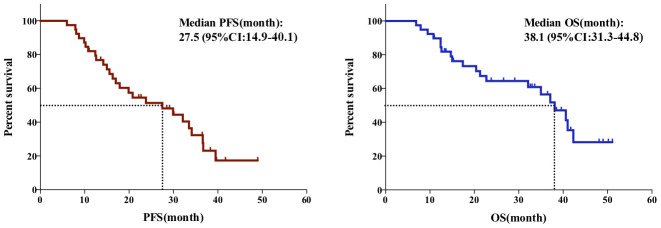
**The PFS (Left) and OS (Right) of the whole cohort.**

At the time of the last follow-up, 19 patients survived from the disease, while 20 died of tumor recurrence or metastases. The median PFS and OS of the whole cohort were 27.5 (95%CI: 14.9-40.1) and 38.1 (95%CI: 31.3-44.8) months, respectively. The Kaplan–Meier curves showed 1- and 3-year PFS rates of 82.1% and 32.3%, respectively. The OS rates at 1-, 3-, and 5-years of all enrolled patients were 89.7%, 56.5%, and 28.3%, respectively (Figure 1).

**Figure 2:**
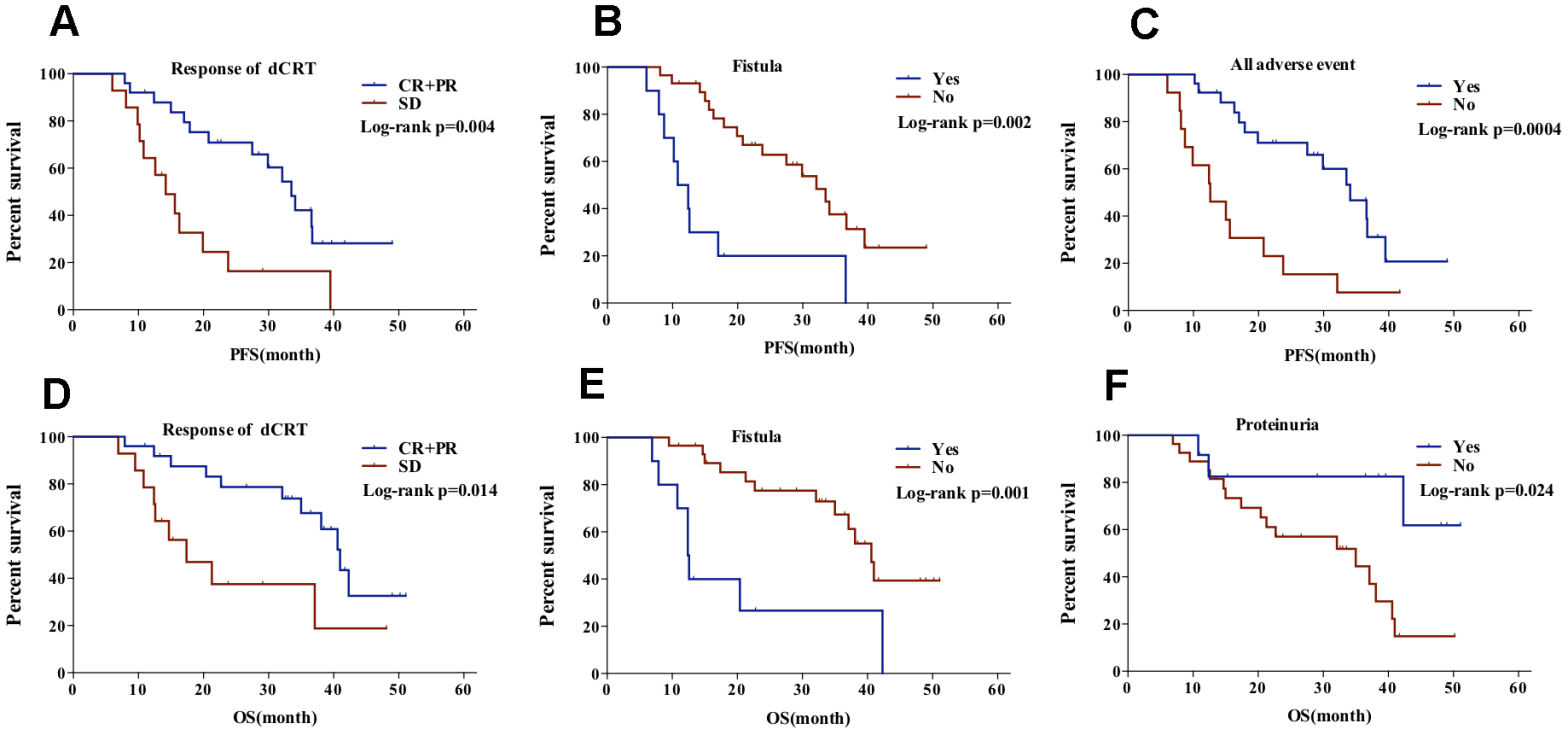
PFS of patients (**A**) with CR+PR vs SD after dCRT, (**B**) with vs without fistula, and (**C**) with vs without any adverse event; OS of patients with (**D**) CR+PR vs SD after dCRT, (**E**) with vs without fistula, and (**F**) with vs without proteinuria.

Univariate analysis showed a significant correlation between poor PFS and variables including SD after dCRT, combination of fistula, no adverse event, no absence of secondary hypertension or hand-foot syndrome (p<0.05). Upon inclusion of these variables in the Cox multivariate regression model, the multivariate analysis showed response after dCRT (HR = 3.663, 95%[CI] = 1.560 - 8.599), combination of fistula (HR = 5.799, 95%[CI] = 2.247-14.961), adverse events (HR = 0.274, 95%[CI] = 0.119 - 0.630), to be independent predictors of PFS in the whole cohort (Figure 2A–2C) (Table 4). Further, multivariate analysis showed SD after dCRT (HR= 5.858, 95%[CI]= 2.082-16.488), combination of fistula (HR= 10.193, 95%[CI]= 3.291 - 31.568), and no absence of proteinuria (HR= 0.108, 95%[CI]= 0.025 - 0.456), predicted worse prognosis in OS (Figure 2D, 2E) (Table 5). No significant between-group difference was found upon treatment with S1/capecitabine followed by apatinib and concurrent treatment with apatinib.

**Table 4:** Univariate and multivariate analysis of PFS.

**Variables**	**univariate analysis**			**multivariate analysis**
	**HR(95% CI)**	**p value**	**log-rank p**	**HR(95% CI)**	**P value**
Gender (male vs female)	0.577(0.169-1.965)	0.379	0.373		
Age(<60 vs ≥60)	0.905(0.417-1.963)	0.8	0.8		
Length(<5 vs ≥5cm)	1.752(0.758-4.051)	0.19	0.184		
TNM stage (II vs III)	2.824(0.664-12.003)	0.16	0.142		
Concurrent Chemotherapy regimens					
Single reagent	Reference	0.105	0.086		
TP	0.330(0.097-1.130)	0.077			
PF	0.356(0.130-0.977)	0.045			
Radiation dose (≤60 vs >60Gy)	0.596(0.249-1.425)	0.244	0.239		
Response (PR vs SD)	3.221(1.448-7.165)	0.004*	0.003*	3.663(1.560-8.599)	0.003*
Residual disease sites					
Primary lesion only	reference	0.779	0.778		
Regional lymph nodes only	0.702(0.251-1.966)	0.501			
Primary lesion combined with Regional lymph nodes	0.898(0.340-2.367)	0.827			
Pathological residual disease (Yes vs No)	2.061(0.939-4.524)	0.071			
Fistula (Yes vs No)	4.149(1.766-9.749)	0.001*	0.0004*	5.799(2.247-14.961)	0.0003*
S1/capecitabine followed by apatinib vs concurrent with apatinib	2.887(0.850-4.191)	0.119	0.113		
side effects related					
Overall side effects (No vs Yes)	0.294(0.134-0.644)	0.002*	0.001*	0.274(0.119-0.630)	0.002*
Secondary hypertension (No vs Yes)	0.335(0.133-0.846)	0.021*	0.015*		
Proteinuria (No vs Yes)	0.425(0.167-1.080)	0.072	0.065		
Hand–foot syndrome (No vs Yes)	0.368(0.146-0.928)	0.034*	0.028*		

TP: taxol and platinum; PF: platinum and fluorouracil; PR: partial response; SD: stable disease

**Table 5:** Univariate and multivariate analysis of OS.

**Variables**	**univariate analysis**			**multivariate analysis**	
	**HR(95% CI)**	**P value**	**log-rank p**	**HR(95% CI)**	**P value**
Gender (male vs female)	0.792(0.226-2.781)	0.716	0.715		
Age(<60 vs ≥60)	1.060(0.440-2.558)	0.896	0.896		
Length(<5 vs ≥5cm)	2.301(0.830-6.376)	0.109	0.099		
TNM stage (II vs III)	2.564(0.583-11.265)	0.213	0.197		
Concurrent Chemotherapy regimens					
Single reagent	Reference	0.583	0.573		
TP	0.506(0.124-2.066)	0.343			
PF	0.580(0.183-1.843)	0.356			
Radiation dose (≤60 vs >60Gy)	0.562(0.204-1.549)	0.265	0.258		
Response (PR vs SD)	2.844(1.145-7.062)	0.024*	0.019*	5.858(2.082-16.488)	0.001
Failure pattern					
Primary lesion only	Reference	0.767	0.764		
Regional lymph nodes only	0.655(0.210-2.050)	0.468			
Primary lesion combined with Regional lymph nodes	0.784(0.267-2.295)	0.656			
Pathological residual disease(Yes vs No)	2.291(0.948-5.538)	0.066			
Fistula	4.224(1.680-10.620)	0.002*	0.001*	10.193(3.291-31.568)	0.00004
S1/capecitabine followed by apatinib vs concurrent with apatinib	1.318(0.528-3.290)	0.555	0.553		
side effects related					
Overall side effects (No vs Yes)	0.277(0.109-0.703)	0.007*	0.004*		
Secondary hypertension (No vs Yes)	0.475(0.178-1.268)	0.475	0.129		
Proteinuria (No vs Yes)	0.244(0.068-0.870)	0.030*	0.020*	0.108(0.025-0.456)	0.002
Hand–foot syndrome (No vs Yes)	0.459(0.172-1.223)	.0.119	0.111		

TP: taxol and platinum; PF: platinum and fluorouracil; PR: partial response; SD: stable disease.

## DISCUSSION

Despite dCRT being a common curative approach for unresectable locally advanced ESCC, only 15-53% of treated patients will achieve CR upon completion of dCRT [16, 17]. Survival of patients with the clinical non-CR disease after initial CRT was substantially poor compared to those with CR [18]. Recent data shows an overall 3-year PFS of around 45% in ESCC patients who received dCRT indicating that over half of treated patients will develop recurrent disease within three years [19]. Patients with clinical non-CR disease account for a major portion of recurrence cases, suggesting that early control of the persistent disease may lead to survival benefits [20]. However, improved survival achieved through consolidated chemotherapy in ESCC patients after dCRT remains controversial, though the inconsistencies reported may be due to the enrollment of patients with and without CR in these studies [21]. Further, it may suggest the need for consolidative or maintenance therapy only for patients with persistent disease.

Thus far, a consensus treatment approach has not been established for patients with residual lesions. Recently, the addition of individualized multimodal approaches such as salvage surgery, photodynamic therapy, and endoscopic mucosal resection after the local failure of dCRT have been reported to prolong patient survival [16, 22–25]. However, only a small subset of patients is eligible for such local treatments. Further, salvage surgery is associated with increased susceptibility to postoperative complications such as anastomotic leakage due to prior treatment with a relatively high dose of radiation [25]. Conventional treatment strategies in clinical practice include close follow-up and second-line chemotherapy is recommended upon confirmation of disease progression. Thus, intravenous systemic chemotherapy is frequently used to arrest disease recurrence and progression. However, patient compliance is generally poor due to the occurrence of high toxicity leading to early termination [6]. Oral drugs include the TKIs and chemotherapy agents are widely used as the substitute for intravenous infusion chemotherapy regimens, suggesting they are also good candidates as the maintenance therapy for those with residual disease after dCRT.

Fluorouracil is a standard chemotherapeutic agent for ESCC. There are now two commercially available oral fluorouracil agents — S-1 and capecitabine — with similar proven anticancer effects compared to intravenous 5-Fu [7, 8]. The safety and efficacy of chemoradiotherapy with concurrent S-1 and cisplatin for ESCC were explored in a phase I/II trial (JCOG0604), which showed a favorable 3-year OS rate of 61.9%, CR rate of 59.5% and, acceptable toxicity, comparable to conventional chemotherapy [26]. Similarly, another phase II study demonstrated that concurrent selective lymph node radiotherapy and S-1 with cisplatin were feasible and well-tolerated in patients with stage II–IVa ESCC [11]. Besides its application as a first-line treatment, S-1 combined with chemotherapy has also been used as a second-line treatment in ESCC [10, 27]. To further improve its advantages as an oral formulation and its convenience as a continuous delivery application, without the need for intravenous infusion compared with 5-Fu, emerging studies have been conducted by combining S-1 with TKIs as dual oral agents.

Apatinib is a small-molecule TKI that targets VEGFR-2, thereby inhibiting VEGF-mediated endothelial cell migration, which impairs tumor microvasculature and suppressing tumor growth [28]. Apatinib was first shown to significantly improve OS and PFS with limited toxicity when used as a later line therapy in advanced adenocarcinoma of the stomach or gastroesophageal junction in a randomized phase III trial [29]. It was then widely applied in gastric cancer and then expanded to the treatment of lung, sarcoma, and liver cancer [30]. The use of apatinib as a second-line treatment monotherapy for advanced ESCC achieved median PFS and OS of 3.5 and 7 months, respectively [13]. These findings were confirmed in a phase II study, which showed that the monotherapy achieved an OR of 7.7%, with median PFS and OS at 4.63 and 6.57 months, respectively [31]. Moreover, *in vivo* and *in vitro* studies have demonstrated that apatinib significantly increased the sensitivity to paclitaxel, cisplatin, and 5-Fu, suggesting that it is feasible to combine apatinib with other chemotherapeutic agents to yield a synergistic effect [32–34].

When combined with S-1, treatment of advanced gastric cancer with apatinib has been explored [35]. Zhao et al. first reported the use of this combination regimen as second-line treatment in 15 advanced ESCC patients who received apatinib (250-500 mg) plus S-1 until disease progression. The median PFS and OS were 6.23 and 8.83 months, respectively, which are longer than previously reported data of monotherapy, suggesting an improvement in the efficacy of tumor control with dual treatment [15]. The results of our retrospective analysis of 39 ESCC patients with residual disease after dCRT who received apatinib plus S-1/capecitabine showed a satisfactory survival with a median PFS of 27.5 (95%CI: 14.9-40.1) and OS of 38.1 (95%CI: 31.3-44.8) months. The reported median OS and OS rate at 3-years of those patients with local advanced ESCC who received dCRT varied from 9.0 to 41.0 months and 21 to 45% [36–38]. Similar to published studies, we found that the response after dCRT, as an independent factor, was correlated with the better prognosis of patients in the multivariate analysis. In those patients who achieved clinical CR or good response, the median OS was 46 months and the 3-years survival rate was 57%, which were significantly better than others [39]. The survival data reported in this study was comparable to the patients who had CR after dCRT, suggesting that maintenance therapy for those non-CR ESCC is a promising strategy to improve survival.

Secondary hypertension, hand-foot syndrome, and proteinuria were the most common adverse events associated with treatment. These largely overlapped with the toxicity profile of anti-angiogenic agents including apatinib, and consistent with previous reports, were grade 1 to 2 in severity and therefore, tolerable [40, 41]. Although one confirmed case showed grade 4 proteinuria, the patient had recovered rapidly upon the cessation of apatinib. In addition, there was no worsening in toxicity when apatinib was combined with S-1/capecitabine, which may reflect the differences in the toxicity spectrum between S-1/capecitabine and apatinib. Similar to other TKIs, toxicity related to apatinib was a predictive biomarker for treatment response and survival [42]. In our multivariate analysis, patients with an occurrence of adverse events related to apatinib showed significantly longer PFS compared to those with none. Moreover, proteinuria was also significantly correlated with improved OS. VEGF-TKI targets VEGFR2 and then decreased the release of nitric oxide from endothelial cells, leading to the constant contraction of arterial smooth muscle cells to induce hypertension [43]. These side effects mostly are caused by the sufficient action of VEGF-TKIs thus representing the ‘‘on-target’’ effect in normal tissues. This partially explained why there was a positive correlation between the occurrence of side effects and better tumor control. However, due to the limited number of enrolled patients, our conclusions must remain tentative.

### Strengths and limitations

To the best of our knowledge, this is the first retrospective analysis to show the use of apatinib combined with S-1/capecitabine as the maintenance therapy for ESCC with residual disease after dCRT. Ongoing prospective clinical trials exploring the efficiency of apatinib alone or in combination with other agents for EC are listed in Table 6. Since apatinib and S-1/capecitabine are orally administered drugs, the convenience of its application offers a significant advantage over other regimens. Further, an improvement in patient compliance to oral therapy is expected since hospital admission or continuous intravenous infusion can be avoided and therefore achieve tumor control over a longer period. The small sample size and the retrospective nature of this study are significant limitations.

**Table 6:** Clinical trials of apatinib in EC.

**Agents**	**Other agents**	**Conditions**	**Phase**	**No.**	**Design**	**Endpoints**	**NCT ID**	**Status**
Apatinib		Metastatic EC	2	40	Randomized; open-label	PFS	NCT02683655	Unknown
	N/A	Metastatic EC	2	29	Single group; open-label	response; AEs; OS; PFS	NCT02544737	Unknown
		Esophageal and Gastric Cancer	2/3	30	Single group; open-label	PFS; OS	NCT03285906	Unknown
		Advanced EC	2	50	Single group; open-label	PFS; OS	NCT03542422	Unknown
		Advanced EC	2	60	Single group; open-label	PFS; OS	NCT03170310	Unknown
		Recurrent and Metastatic EC	2	40	Single group; open-label	PFS; OS; response	NCT03274011	Active, not recruiting
		Recurrent and Metastatic EC	2	39	Single group; open-label	PFS; OS; response	NCT03913182	Recruiting
		Multiple Malignancies including EC	4	38	Single group; open-label	response; AEs; OS; PFS	NCT03384511	Completed
		Recurrent and Metastatic EC	2	120	Randomized; parallel; single-blind	PFS; OS; response	NCT03787251	Not yet recruiting
		EC	2	43	Single group; open-label	response; AEs; OS; PFS	NCT02976896	Recruiting
	Irinotecan	Unresectable or Metastatic EC	2	50	Single group; open-label	response; AEs; OS; PFS	NCT03251417	Recruiting
		EC	1	9	Single group; open-label	response; AEs; OS; PFS	NCT02645864	Unknown
	Docetaxel	Advanced EC	2	120	Randomized;parallel Assignment	PFS; OS	NCT03193424	Unknown
	Docetaxel, Nedaplatin, Endostar VS Docetaxel, Nedaplatin	EC	2	186	Randomized; parallel; single-blind	PFS; OS	NCT03649945	Not yet recruiting
	Fluorouracil and platinum	EC	2	189	Randomized; parallel	response; AEs; OS; PFS	NCT03224221	Unknown
	Paclitaxel, Cisplatin+RT	EC	2	40	Single group; open-label	response; AEs; OS; DFS	NCT03857763	Not yet recruiting
	S-1+ RT	Refractory or Metastatic EC	2	80	Randomized; parallel; open-label	response; AEs; OS; DFS	NCT03320629	Unknown
	SHR-1210	Advanced EC	2	45	Single group; open-label	PFS; response	NCT03736863	Not yet recruiting
	SHR-1210+ Docetaxel, Cisplatin+ RT	Local Advanced EC	NA	20	Single group; open-label	response; AEs; OS; PFS	NCT03671265	Recruiting
	SHR-1210+ Irinotecan, Paclitaxel, Nedaplatin	Advanced EC	2	45	Non-Randomized; parallel; open-label	response; AEs; OS; PFS	NCT03603756	Recruiting

RT: radiotherapy; EC: esophageal carcinoma; AEs: adverse events; OS: overall survival; PFS: progression-free survival; DFS: disease-free survival.

## CONCLUSIONS

In conclusion, our study suggested that the oral combination therapy of apatinib and S-1/capecitabine holds significant and promising efficacy with manageable toxicity for the treatment of ESCC in patients with residual disease after dCRT. Prospective clinical trials are warranted to further confirm the feasibility of this treatment regimen.

## MATERIALS AND METHODS

This study was approved by the Sun Yat-Sen University Cancer Center Human Research Ethical Committee. Informed consent was obtained from all enrolled patients.

### Eligibility criteria

Patients diagnosed with ESCC and post-dCRT residual lesions were enrolled in this retrospective study. The inclusion criteria were (1) Histopathologically-confirmed ESCC; (2) Age ≥18 years; (3) Eastern Cooperative Oncology Group - Performance Status (ECOG-PS) scores from 0-2; (4) Local residual disease confirmed by endoscopy, ultrasonography, computed tomography (CT), physical examination and/or biopsy at three months after the completion of dCRT; (5) No distant metastasis; (6) Life expectancy ≥ 6 months.

### Treatment regimen

Patients in this retrospective analysis were orally treated with S-1 twice daily at 40 mg (body surface area <1.25 m^2^) or 60 mg (body surface area ≥ 1.25 m^2^). In the event of shortage, unacceptable toxicity, or patient refusal of S-1, capecitabine was orally administered twice daily at 1.25g/m^2^. Treatment with S-1 or capecitabine treatment followed a four-week cycle consisting of three weeks of treatment followed by one-week rest. Patients were concurrently treated with apatinib once daily at 250 to 500 mg per four-week cycle. Patients who could not tolerate dual treatments at the beginning were treated initially with S-1/capecitabine and sequentially with apatinib after 1-2 cycle of S-1/capecitabine. Treatments were scheduled until the progressive disease was confirmed, due to unacceptable toxicity or patient refusal.

### Response, toxicity, and survival

Patient’s response to treatment was assessed by CT scan and endoscopy at one or two months after initiating maintenance therapy, according to Response Evaluation Criteria in Solid Tumors 1.1 (RECIST 1.1) criteria. The ORR and DCR were calculated. During treatment, complete blood count, serum chemistry profile, and urine routine test were examined each month to monitor toxicity. Adverse events were assessed and graded into 0-V degrees according to the Common Terminology Criteria for Adverse Events version 3.0 (CTCAE 3.0). The duration from maintenance treatment to tumor progression or death (Progression-Free Survival; PFS), and to mortality or the last follow-up (Overall Survival; OS) were calculated.

### Statistical analysis

The Kaplan–Meier curves with the log-rank test were used to analyze and compare the median PFS and OS. Multivariate analysis was performed using Cox regression to analyze the prognostic factors. The SPSS 26.0 and GraphPad Prism 5.0 were used for analyses. P <0.05 considered being statistically significant.
